# Curcumin and o-Vanillin Exhibit Evidence of Senolytic Activity in Human IVD Cells In Vitro

**DOI:** 10.3390/jcm8040433

**Published:** 2019-03-29

**Authors:** Hosni Cherif, Daniel G. Bisson, Peter Jarzem, Michael Weber, Jean A. Ouellet, Lisbet Haglund

**Affiliations:** 1 Orthopaedic Research Lab, Department of Surgery, McGill University, 1650 Cedar Ave., C10-148.8, Montreal, QC H3G 1A4, Canada; hosni.cherif@hotmail.com (H.C.); daniel.g.bisson@gmail.com (D.G.B.); 2 McGill Scoliosis and Spine Group, Department of Surgery, McGill University, 1650 Cedar Ave., C10-148.8, Montreal, QC H3G 1A4, Canada; pjarzem@gmail.com (P.J.); micheal.weber@hotmail.com (M.W.); jaouellet@hotmail.com (J.A.O.); 3 Department of Surgery, The Research Institute of the McGill University Health Centre, 1650 Cedar Ave., C10-148.8, Montreal, QC H3G 1A4, Canada; 4 Shriner’s Hospital for Children, 1003 Decarie Blvd, Montreal, QC H4A 0A9, Canada

**Keywords:** intervertebral disc, senescence, degeneration, inflammation, senolytics, back pain

## Abstract

Curcumin and o-Vanillin cleared senescent intervertebral disc (IVD) cells and reduced the senescence-associated secretory phenotype (SASP) associated with inflammation and back pain. Cells from degenerate and non-mildly-degenerate human IVD were obtained from organ donors and from patients undergoing surgery for low back pain. Gene expression of senescence and SASP markers was evaluated by RT-qPCR in isolated cells, and protein expression of senescence, proliferation, and apoptotic markers was evaluated by immunocytochemistry (ICC). The expression levels of SASP factors were evaluated by enzyme-linked immunosorbent assay (ELISA). Matrix synthesis was verified with safranin-O staining and the Dimethyl-Methylene Blue Assay for proteoglycan content. Western blotting and ICC were used to determine the molecular pathways targeted by the drugs. We found a 40% higher level of senescent cells in degenerate compared to non-mildly-degenerate discs from unrelated individuals and a 10% higher level in degenerate compared to non-mildly-degenerate discs from the same individual. Higher levels of senescence were associated with increased SASP. Both drugs cleared senescent cells, and treatment increased the number of proliferating as well as apoptotic cells in cultures from degenerate IVDs. The expression of SASP factors was decreased, and matrix synthesis increased following treatment. These effects were mediated through the Nrf2 and NFkB pathways.

## 1. Introduction

Back pain is a leading cause of disability and loss of workplace productivity in otherwise healthy young and middle-aged individuals. IVD degeneration is widely recognized as a contributor to low back pain, and several factors such as age, genetic predisposition, and mechanical stress have been suggested to contribute to the initiation and progression of IVD degeneration [1]. The degenerating IVD constitutes an inflammatory environment, with the accumulation of senescent cells. Cell senescence is a durable cell-cycle arrest that is triggered by a myriad of extracellular and intracellular stimuli [2]. Stress-induced premature senescence can be triggered by DNA damage, oxidative stress, adverse load, and inflammation [3]. Senescent cells, although permanently non-dividing, remain metabolically active and secrete a range of pro-inflammatory cytokines, chemokines, proteases, and growth factors, called the SASP. The SASP influences the tissue microenvironment, accelerates aging, and contributes to local and systemic dysfunction in diseases such as osteoarthritis [4,5]. Furthermore, it can, in a paracrine manner, induce senescence, which further intensifies tissue deterioration [6]. Several markers have been used to identify senescent cells. Activation of the lysosomal enzyme senescence-associated β-galactosidase (SA-β-gal) was the first reliable indicator of senescent cells. More specific molecular biomarkers, such as the cyclin-dependent kinase inhibitor 2A (also known as p16*^INK4a^*), are now also used [7].

Drugs are emerging that either kill (senolytic) or reverse cellular senescence (senomorphic). Clinically approved senolytic drugs, including glucocorticoids, metformin, and rapamycin inhibitors, can restore tissue homeostasis [2], and recent data suggest that they can prevent or even treat cartilage-related diseases like sarcopenia, osteoarthritis, kyphosis, and herniated discs [4]. Examples of senomorphic compounds, include free radical scavengers and inhibitors of IkB kinase (IKK), nuclear factor (NFkB) [8] and the Janus kinase (JAK) pathways [9]. These drugs suppress markers of senescence or their secretory phenotype without inducing apoptosis. Some compounds can be senolytic and/or senomorphic, depending on the cell type. For example, fisetin is a natural compound that has senomorphic effects on senescent human lung fibroblasts (IMR90) or pre-adipocytes, and selectively kills IR-induced senescent but non-proliferating human umbilical vein endothelial cells [10,11].

One of the compounds of interest in this study, curcumin (diferuloylmethane), has therapeutic benefits via its antioxidant and anti-inflammatory properties [12]. It has been shown to exert cytotoxic effects on cancer cells without being toxic to non-malignant cells [13]. However, curcumin has poor bioavailability, low water solubility, and chemical instability. An increase in interest has therefore been raised with regard to the biological properties of curcumin’s metabolites [14]. o-Vanillin (2-hydroxy-3-methoxybenzaldehyde) is the main metabolite, and like curcumin, it has documented anti-inflammatory and antioxidative properties [15] and is present in high concentrations in circulation after curcumin consumption [16]. Both compounds interfere with multiple cellular processes, including cell proliferation, cell survival and apoptosis [17,18]. To the best of our knowledge, no study has yet evaluated the potential senolytic and/or senomorphic effects of curcumin and o-Vanillin on human IVD cells.

## 2. Experimental Section

### 2.1. Study Approval and Tissue Collection

Collection of human disc tissue was approved by the ethical review board at McGill University (IRB#s A04-M53-08B and A10-M113-13B). The tissue demographics are presented in Table 1. Nucleus pulposus (NP) and annulus fibrosis (AF) cells were isolated separately, as previously described [19]. Briefly, NP and AF tissue were separated microscopically according to their different macroscopic morphologies. Next, the samples were washed in phosphate-buffered saline solution (PBS, Sigma-Aldrich, Oakville, ON, Canada) and Hank’s-buffered saline solution (HBSS, Sigma-Aldrich, Oakville, ON, Canada) supplemented with Primocin^TM^ (InvivoGen, San Diego, CA, USA) and Fungiozone (Sigma-Aldrich, Oakville, ON, Canada). Then, the matrix was minced and digested in 0.15% collagenase type II (Gibco) for 16 h at 37 °C. Cells were passed through a 70-μm filter and re-suspended in DMEM (Sigma-Aldrich, Oakville, ON, Canada) supplemented with 10% fetal bovine serum (FBS (Gibco)), Primocin^TM^ (100 mM), Glutamax (1X) (Sigma-Aldrich, Oakville, ON, Canada), and maintained in a 5% CO_2_ incubator at 37 °C.

### 2.2. In Vitro Disc Cell Cultures

Experiments were performed with NP and AF separately. Cells were used directly following digestion for pellet cultures and within passages 0–1 for monolayer cultures.

Monolayer culture: 20,000 or 250,000 cells per well were seeded in 8-well chamber slides (Nunc™ Lab-Tek™ II Chamber Slide™ System) and 6-well plates (Sarstedt, TC plate 6-well, Cell+, F) respectively. Cells were serum-starved in DMEM with ITS (1X) (Thermo Fisher, Waltham, MA, USA) for 2 h prior to treatment with 5 μM curcumin (Sigma-Aldrich, Oakville, ON, Canada), 100 μM o-Vanillin (Sigma-Aldrich, Oakville, ON, Canada), 50 μM peroxide (Sigma-Aldrich, Oakville, ON, Canada), or vehicle (DMSO (0.01%, (Sigma-Aldrich, Oakville, ON, Canada) for 1 and 6 h.

Pellet culture: 300,000 NP cells/tube were collected by centrifugation at 500× *g* for 5 min. Pellets were incubated in 1 mL DMEM (2.25 g/L glucose, 5% FBS, ascorbic acid (5 μM) (Sigma-Aldrich, Oakville, ON, Canada)) at 37 °C and 5% CO_2_. Culture media was changed every 3 days. Cell pellets were washed in PBS, cryoprotected in 10–30% sucrose, then embedded in Optimum Cutting Temperature compound (OTC, Thermo Fisher, Waltham, MA, USA), and finally flash-frozen and kept at −80 °C. Sections 5-µm-thick were cut with a cryostat (Leica Microsystems, Richmond Hill, ON, Canada) and placed on slides for immunostaining.

### 2.3. SA-β-Gal Staining

SA-β-gal staining was carried out on cells that were seeded in 8-well chamber slides, according to the manufacturer’s protocol (Sigma-Aldrich, Oakville, ON, Canada). Following treatment, the culture media was removed, and the cells were washed twice with PBS, then fixed with the provided fixation buffer for 10 min at room temperature. After rinsing three times with PBS, the staining mixture was added, and the plate was sealed with Parafilm and incubated overnight at 37 °C. Coverslips were mounted using Aqua Polymount (Polysciences, Warrington, PA, USA), and slides were visualized using the Zeiss Axioskop40 microscope and an AxioCam MR camera. Images were processed using AxioVision LE64 software (Zeiss, Oberkochen, Germany). Ten fields, randomly distributed across the well, were analyzed, and the number of positive (blue stained) and total cells were counted, and the percentage of senescent cells was calculated.

### 2.4. Immunochemistry

Monolayer cultures were washed with PBS, fixed with 4% paraformaldehyde (Thermo Fisher, Waltham, MA, USA), and blocked in PBS with 1% BSA (Sigma-Aldrich, Oakville, ON, Canada), 1% serum, and 0.1% Triton X-100 (Sigma-Aldrich, Oakville, ON, Canada) for 1 h. Slides were then incubated with the p16*^INK4a^* CINtec PLUS Kit (Roche, Ventana laboratories, Mississauga, ON, Canada) according to the manufacturer’s instructions. Slides were also exposed to primary antibodies specific to Ki-67 (Novus, Oakville, ON, Canada), caspase 3 (Sigma-Aldrich, Oakville, ON, Canada), Nrf2, or p65 (Cell signalling) overnight at 4 °C. After washing, slides were incubated with the appropriate Alexa Fluor^®^ 488-conjugated secondary antibody (Thermo Fisher, Waltham, MA, USA) for 1 h at room temperature, and then counterstained with DAPI for p65 and Nrf2 immunofluorescence. A mouse- and rabbit-specific HRP/DAB (ABC) Detection IHC Kit (ab64264, Abcam, Cambridge, Ma, USA) was used for caspase-3 and Ki-67 staining, and counterstaining was performed by Mayer’s hematoxylin (Sigma-Aldrich, Oakville, ON, Canada). Apoptosis was detected using a commercial kit (ab176749, Abcam, Cambridge, Ma, USA) according to the manufacturer’s instructions. Photomicrographs were acquired with a fluorescent Olympus BX51 microscope equipped with an Olympus DP71 digital camera (Olympus, Tokyo, Japan). Bright-field images were acquired as described for SA-β-gal staining.

Pellet sections were heated at 60 °C for 30 min, then washed in PBS, and fixed in 4% paraformaldehyde. Cells were permeabilized with 0.3% Triton X-100 in PBS, saturated with 1% BSA, 1% serum, and 0.1% Triton X-100 for 1 h, and then incubated overnight at 4 °C for p16*^INK4a^* and 1 h at room temperature for Ki-67 and caspase-3 primary antibodies. The HRP/DAB Detection IHC Kit was used for detection. Samples were also stained with Safranin-O (Sigma-Aldrich, Oakville, ON, Canada) and antibodies to collagen type II (ab34712, Abcam, Cambridge, Ma, USA). Regarding p16*^INK4a^* detection, only nuclear and nuclear with cytoplasmic immunostaining were considered positive [20,21]. Coverslips were mounted using Aqua Polymount, and images were captured as described for SA-β-gal and analyzed with ImageJ and MatLab script [22].

### 2.5. Metabolic Activity

Metabolic activity was assessed by the Alamar Blue assay [23]. Briefly, NP and AF cells (1 × 10^4^) were seeded in 96-well tissue culture plates for 12 h prior to exposure to 0, 5, 10, 20, 30, 40, 50, 75, 100, or 200 μM curcumin and o-Vanillin, for 6 h. After exposure, 10% Alamar Blue reagent (Thermo Fisher, Waltham, MA, USA) was added to each well and incubated for 4 h at 37 °C. Fluorescence (Ex560/Em590) was measured by using a spectrophotometer (Tecan Infinite T200, Männedorf, Switzerland) equipped with Magellan software (Tecan, Männedorf, Switzerland). Results are presented as a percentage of metabolic activity compared to the control. Experiments were performed (3–6) times in triplicate wells for each compound and concentration.

### 2.6. Caspase 3/7 Activity Assay

After treatment, the caspase 3/7 activity of degenerate and non-mildly-degenerate NP cells was measured using the Amplite Fluorimetric Caspase 3/7 Assay Kit (AAT Bioquest, Sunnyvale, CA, USA) as per the manufacturer’s protocol. Briefly, cells were incubated with the caspase 3/7 assay solution, which contained caspase substrate (Z-DEVD-R110), at room temperature for 1 h in the dark. Fluorescence intensity was then measured at 490 nm excitation and 525 nm emission. The results are expressed as a percentage of the mean of the control group (set at 100%). Each experiment was performed in triplicate and carried out three times from each round of cell isolation.

### 2.7. RT-qPCR

Following treatment, RNA was extracted using the TRIzol chloroform extraction method, as previously described [24]. Briefly, 500 ng of RNA was reverse-transcribed using a qScript cDNA Synthesis Kit (Quanta Biosciences, Beverly, MA, USA) with an Applied Biosystems Verti Thermocycler (Thermo Fisher, Waltham, MA, USA). RT-qPCR was performed using an Applied Biosystems StepOnePlus machine (Thermo Fisher, Waltham, MA, USA) with PerfecCTa SYBR Green Fast Mix (Quanta Biosciences, Beverly, MA, USA). Previously published primers for senescence, inflammatory markers, and housekeeping genes are described in Table 2. All reactions were conducted in triplicate, and fold-changes in gene expression were calculated by using the 2^−ΔΔCt^ method [25], after normalizing to the housekeeping gene and vehicle-treated cells.

### 2.8. ELISA

SASP factors (IL6, IL8, MMP3 and MMP13) in 100 μL pooled cell pellet culture media from day 0 through to day 21 were determined using ELISA kits, according to the manufacturer’s instructions (RayBiotech, Norcoss, GA, USA). Colorimetric absorbance was measured with a Tecan Infinite M200 PRO (Tecan, Männedorf, Switzerland) spectrophotometer and analyzed with i-control 1.9 Magellan software (Tecan, Männedorf, Switzerland). Protein levels of the treated conditions and the vehicle control were then compared.

### 2.9. DMMB

DMMB assays to quantify sulfated glycosaminoglycans (sGAG) in the media of degenerate NP pellets with or without curcumin or o-Vanillin treatment were performed as previously described [27]. Chondroitin sulfate was used to generate the standard curve. Conditioned media samples were pooled from days 4 to 21. All samples were diluted to fall within the middle of the linear portion of the standard curve, then placed in triplicate into clear 96-well plates (Costar, Corning, NY, USA); later, DMMB dye was added to the wells. Samples were analyzed for absorbance at room temperature immediately after adding DMMB dye, using a spectrophotometer (Tecan Infinite T200, Männedorf, Switzerland).

### 2.10. Western Blot Analysis

Following treatment, cells were washed once with ice-cold PBS (pH 7.4) and then lysed in hot Laemmli sample buffer. Twenty micrograms of protein/sample of the homogenate was resolved with 10% SDS-polyacrylamide gel electrophoresis, transferred onto a nitrocellulose membrane, blocked with 5% skim milk, and incubated overnight with antibodies recognizing total or phosphorylated AKT, ERK1/2, JNK, Nrf2, p38, p65, and β-actin (Cell Signalling, Beverly, MA, USA). HRP-coupled secondary antibodies (Abcam, Cambridge, Ma, USA) were used, and detection was performed using Western Lightning Plus (NEL103E001EA, PerkinElmer, Woodbridge, ON, Canada). The intensity of each band was normalized to that of β-actin, and the data are presented as relative intensity.

### 2.11. Statistical Analysis

The data was analyzed using Graph Prism 6 (Graph Pad, La Jolla, CA, USA). A paired t-test was used to analyze the two groups, and a multiple pairwise comparison (Analysis of Variance (ANOVA) was used to evaluate the variance with Bonferroni’s post hoc test. The significance was set at *p* < 0.05.

## 3. Results

### 3.1. Senescent Human IVD Cells Increase with the Degree of Degeneration

**Figure 1:**
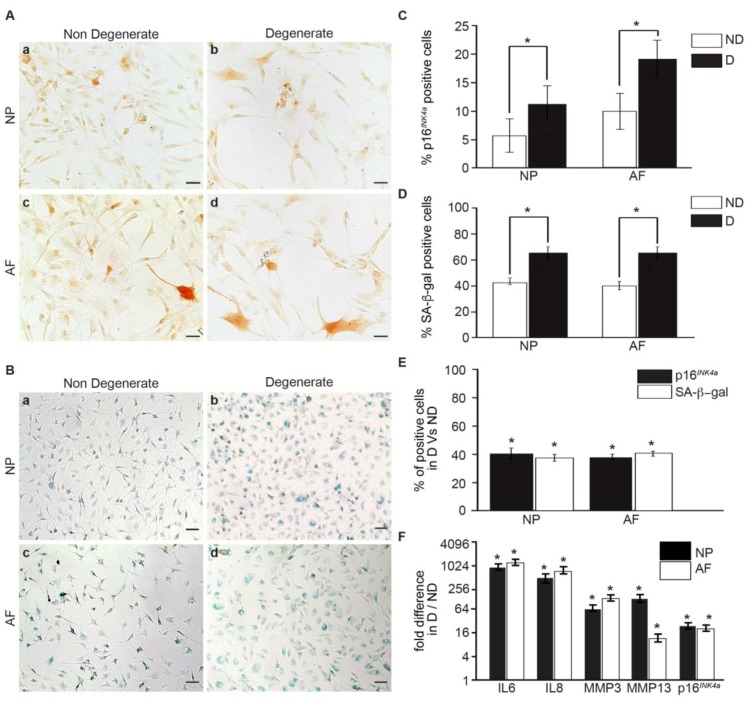
Degenerate NP and AF cells isolated from patients undergoing surgery for low back pain express a higher level of senescent cells and SASP factors, compared to cells isolated from non-mildly-degenerate discs. (**A**) Representative photomicrographs of p16*^INK4a^* expression and (**B**) SA-β-gal activity in NP and AF cells isolated from surgical samples (degenerate: **D**), and non-mildly-degenerate discs (ND). Quantification of (**C**) p16*^INK4a^* and (**D**) SA-β-gal-positive NP and AF cells. (**E**) Comparison of senescent cells in degenerate compared to non-mildly-degenerate NP and AF cells, as detected by both markers. (**F**) Relative mRNA expression of p16*^INK4a^* and SASP genes in degenerate compared to non-mildly-degenerate cells. (*n* = 13: 7—non-mildly-degenerate (average age = 40.42 ± 17.23) and six degenerate (Average age = 33 ± 4.09)). Scale bars: 25 μm (**A**) and 50 μm (**B**). Values are presented as mean ± SEM. * indicates a significant change; *p* < 0.05.

To investigate the contribution of degeneration in human IVD cell senescence, NP and AF cells were obtained from patients undergoing spinal fusion or disc replacement surgery for chronic LBP (degenerate, average age, 33), or from organ donors (non-mildly-degenerate, average age, 40). In the initial experiments, we sought to determine culture conditions with minimal contribution to induced senescence. IVD cells are accustomed to low glucose levels in vivo, but standard in vitro culture media often contains high glucose levels. We compared two commonly used glucose levels (4.5 g/L and 2.25 g/L) and discovered that the percentage of p16*^INK4a^* positive cells was significantly higher in the high-glucose group (4.5 g/L) compared with the low-glucose group (2.25 g/L) for both the NP and AF cells. Thus, we used low-glucose media in all subsequent experiments (Figure S1). The number of senescent cells was compared by using two senescent markers: the expression of p16*^INK^*^4*a*^ protein and the measure of SA-β-gal activity. Both methods detected a higher number of senescent cells from degenerate IVDs (Figure 1A,B). A total of 11% of cells from degenerate and 6% of cells from non-mildly-degenerate NP tissue were positive, whereas 18% of the cells from degenerate and 10% of cells from non-mildly-degenerate AF tissues stained positive for p16*^INK^*^4*a*^ (*p* < 0.05) (Figure 1C). A total of 69% of cells from degenerate and 43% of cells from non-mildly-degenerate NP tissue, compared to 69% of cells from degenerate and 40% of cells from non-mildly-degenerate AF tissue, stained positive for SA-β-gal (*p* < 0.05) (Figure 1D). Regardless of the differences in the exact numbers of cells determined as senescent, both methods showed a 40% higher number of senescent cells in degenerate AF and NP tissue when compared to the corresponding non-degenerate tissues (*p* < 0.05) (Figure 1E). Hydrogen peroxide exposure [28] was used as a positive control, and both methods detected a strong increase in senescent cells following hydrogen peroxide treatment (Figure S2). We then assessed the gene expression of the senescence marker p16*^INK^*^4*a*^ and SASP factors in cells from degenerate and non-mildly-degenerate IVD tissues. We found that p16*^INK^^4a^* (17.78-fold), IL6 (929.77-fold), IL8 (603.35-fold), MMP3 (78.43-fold), and MMP13 (23.98-fold) were significantly higher in cells from degenerate tissues, compared to non-mildly-degenerate (*p* < 0.05) (Figure 1F). Moreover, the mRNA expression levels of SASP genes were positively correlated with the senescence marker. Collectively, these results suggest a detrimental role of cell senescence in the pathogenesis of IVD degeneration.

### 3.2. Within the Same Individual, Degenerate Discs Have Higher Numbers of Senescent Cells than Non-Mildly-Degenerate Tissues

**Figure 2:**
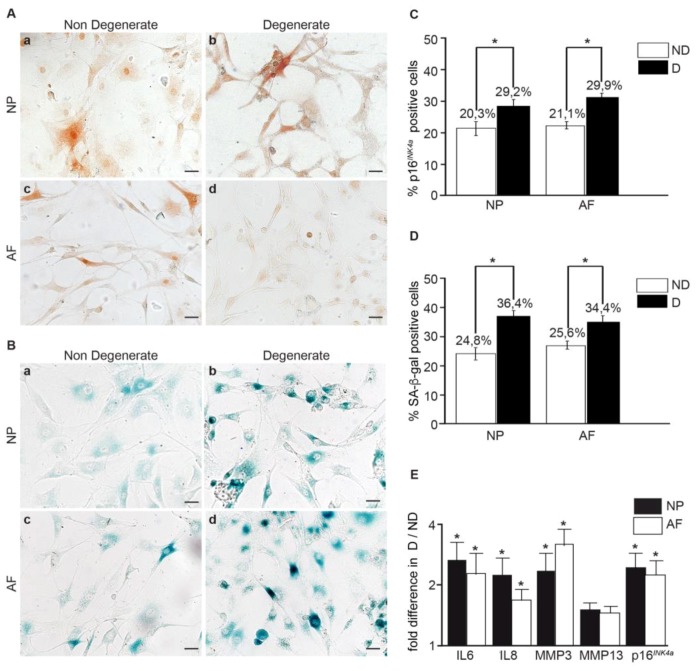
Senescent cells and SASP factor expression in cells from degenerate and non-mildly-degenerate IVDs of the same individual. Representative images of (**A**) p16*^INK4a^* expression and (**B**) SA-β-gal activity in NP and AF cells isolated from degenerate and non-mildly-degenerate discs of the same individual. Quantification of (**C**) p16*^INK4a^* expression and (**D**) SA-β-gal activity. (**E**) Gene expression of SASP factors and p16*^INK4a^* in degenerate AF and NP cells compared with non-mildly-degenerate cells from the same donor. (*n* = 6, Average age = 62 ± 14.68). Scale bars: 25 μm (**A**,**B**). Values are presented as mean ± SEM. * indicates a significant difference; *p* < 0.05.

To better link cellular senescence to IVD degeneration while avoiding confounding factors such as lifestyle, sex, and diseases in specimens from separate individuals, we compared the senescence levels and matrix production in cells from degenerate and non-mildly-degenerate discs of the same individual. The degeneration grade was evaluated by using macroscopic and radiographic scales prior to cell isolation [29,30]. Discs were divided into non-mildly-degenerate (Thompson grade ≤ II) and degenerate (Thompson grade ≥ III) groups (*p* < 0.001) (Figure S3A). Proteoglycan content (Figure S3B(a,b)) and collagen type II (Figure S3B(c,d)) expression were higher in the cell pellets from IVDs with non-mild-degeneration, whereas p16*^INK^*^4*a*^ protein levels and SA-β-gal activity were significantly higher in cell pellets from degenerate IVDs. The level p16*^INK^*^4*a*^-positive cells were 8.9% higher in NP and 8.8% higher in AF cells from degenerate IVDs (*p* < 0.05) (Figure 2A,C), and the number of SA-β-gal-positive cells was 11.6% higher in NP and 8.8% higher in AF cells from degenerate IVDs, compared to the corresponding non-mild-degenerated discs from the same individual (*p* < 0.05) (Figure 2B,C). In addition, a significant two- to three-fold increase in p16*^INK^*^4*a*^, IL6, IL8, and MMP3 (*p* < 0.05) gene expression levels was detected in cells from degenerate IVDs. No change was observed for MMP13 (*p* = 0.15) (Figure 2E). These findings confirm the correlation between the level of cell senescence and SASP factor expression.

### 3.3. The Number of Senescent Cells and the SASP Factor Expression Are Reduced, While Proliferation and Apoptosis Are Increased, Following Curcumin and o-Vanillin Treatment of Degenerate IVD Cells Cultured in a Monolayer

**Figure 3:**
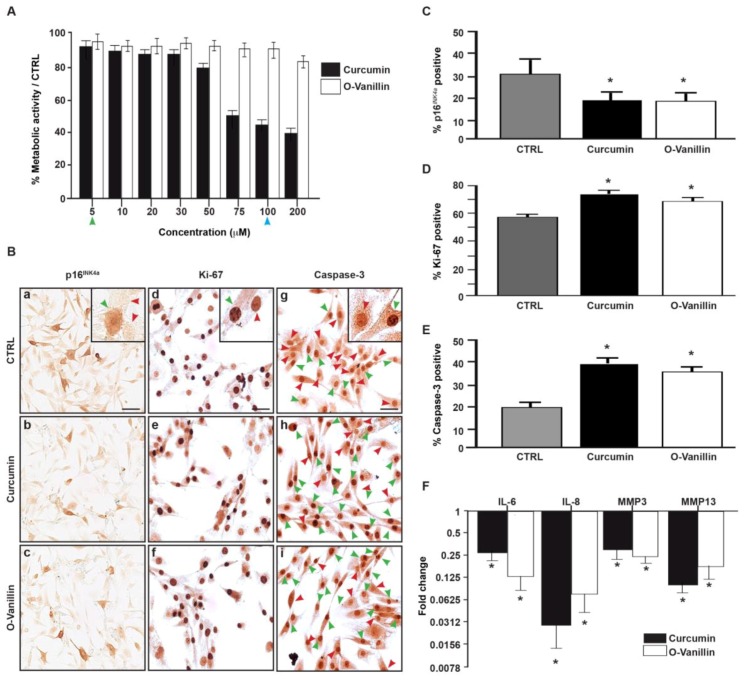
Curcumin and o-Vanillin reduced the number of senescent cells in a monolayer culture. (**A**) The cytotoxicity effects of curcumin and o-Vanillin on degenerated NP (dNP) cells were evaluated by the Alamar Blue assay. Data is presented as a percentage of metabolic activity, compared to untreated cells. Head arrows indicate the concentration that is used for curcumin (green) and o-Vanillin (blue) in subsequent experiments. (**B**) Representative images of p16*^INK4a^* (a–c), Ki-67 (d–f), and caspase-3 (g–i) staining of d-NP cells cultured in a monolayer with or without treatment with curcumin (5 μM) or o-Vanillin (100 μM). Head arrows indicate positive (green) and negative (red) staining. The fragmented nuclear morphology in positive caspase-3 indicate DNA fragmentation in apoptotic cells [31]. Quantification of (**C**) p16*^INK4a^* expression, (**D**) Ki-67, and (**E**) caspase-3. (**F**) Gene expression of the cytokines (IL6, IL8) and proteases (MMP3, MMP13) in d-NP cells treated with curcumin and o-Vanillin, compared to untreated cells (*n* = 6). Scale bars: 10 μm (**A**). Values are presented as mean ± SEM. * indicates a significant difference of; *p* < 0.05.

We used Alamar Blue^®^ to determine a non-toxic concentration range for curcumin and o-Vanillin. Curcumin concentrations between 5 μM and 50 μM did not affect metabolic activity, whereas concentrations of 75–200 µM decreased it. No cytotoxic effect was observed for o-Vanillin at the concentrations tested (*p* = 0.19) (Figure 3A). Based on this and on the concentrations described in the literature, we selected a concentration of 5 μM for curcumin and 100 μM for o-Vanillin for the subsequent experiments. Their potential senolytic and/or senomorphic effects were evaluated in monolayer cultures of NP cells isolated from degenerate IVD. We observed a significant 10% decrease in senescent cells following treatment, as revealed by p16*^INK^^4a^* staining (Figure 3B(a–c)) and quantification (*p* < 0.05) (Figure 3C). The reduction of senescent cells was accompanied with an increase in the cell proliferation of the remaining cells (Figure 3B(d,f)). The number of Ki-67-positive NP cells was significantly increased by 16% following curcumin treatment and by 15% following o-Vanillin treatment (*p* < 0.05) (Figure 3D). Also, both compounds increased in the number of caspase-3-positive apoptotic cells (Figure 3A(g–i)). As shown in Figure 3E, caspase-3-positive cells increased by 18% for curcumin and by 15% for o-Vanillin, compared with the untreated controls (*p* < 0.05). qPCR analysis revealed decreased levels of SASP inflammatory cytokines (IL-6 and -8) and proteases (MMP-3 and -13) in the treated cultures, compared with the untreated control cultures. NP cells had a four-fold IL-6 and 32-fold IL-8 reduction in comparison to untreated cells, while o-Vanillin showed an 8-fold IL-6 and 16-fold IL-8 reduction. The downregulation of MMP-3 (three-fold for curcumin and four-fold for o-Vanillin) and MMP-13 (nine-fold for curcumin and seven-fold for o-Vanillin) in treated cells was comparable for the two drugs (*p* < 0.05) (Figure 3F). To our knowledge, these findings are the first to report a senolytic effect of curcumin and o-Vanillin in human IVD cells.

### 3.4. Increased Proliferation and Beneficial Apoptosis in Degenerate NP Cells Following Treatment Support the Senolytic Activity of Curcumin and o-Vanillin

**Figure 4:**
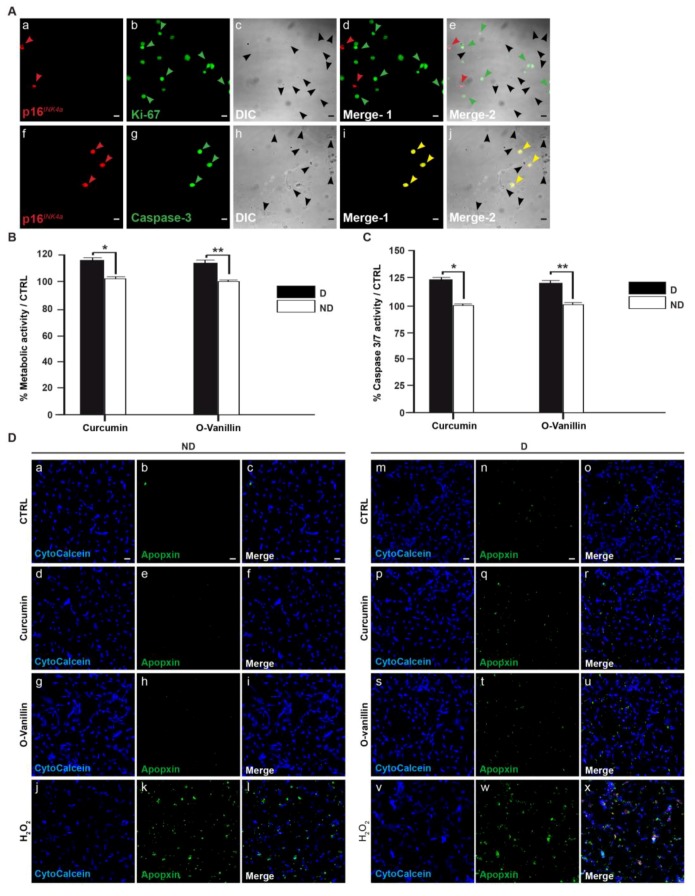
Senolytic activity of Curcumin and o-Vanillin in degenerate but not in non-mildly-degenerate NP cells. (**A**) Double-staining of p16*^INK4a^* and Ki-67 or caspase-3. (a,f) p16*^INK4a^* staining in red. (b,g) Ki-67 and caspase-3-labeled cells in green. (c,h) Differential interference contrast images (DIC). (d,e) Merged images with and without DIC for the double-staining of p16*^INK4a^* and Ki-67. p16*^INK4a^* (red) is not co-localized with Ki-67 (green)-labeled NP cells. (i,j) Merged images with and without DIC for the double-staining of p16*^INK4a^* and caspase-3. p16*^INK4a^* staining (red) largely overlaps with caspase-3 staining (green). (**B**) Comparison of the effects of curcumin and o-Vanillin at the selected concentrations on cell viability in degenerate and non-mildly-degenerate NP cells cultured in a monolayer. Evaluation is with the Alamar blue assay, and presentation is in fold-change compared to the control (*n* = 3). (**C**) Curcumin and o-Vanillin selectively induced apoptosis in degenerate NP cells compared to non-mildly-degenerate as measured by the caspase 3/7 activity kit. The results are expressed as a percentage compared to the untreated control (set at 100%). (**D**) (a–l) Representative photomicrographs of treated and untreated non-mildly-degenerate NP cells stained for cytocalcein (viable) and apopxin (apoptotic), and merged images. (m–x) Representative photomicrographs of treated and untreated degenerate NP cells with the same staining. *n* = 3, scale bars: 50 μm in (**A**,**D**). Values are presented as mean ± SEM in (**B**,**C**). * indicates a significant difference of *p* < 0.05 and ** of *p* < 0.01.

To rule out the idea that the drugs induce apoptosis in non-senescent cells, metabolic activity was compared in cells of degenerate and non-mildly-degenerate NP tissues. The cells were treated with both compounds at the selected concentrations. Using confocal microscopy, double-immunofluorescence staining of p16*^INK^^4a^* with either caspase-3 or Ki-67 markers showed positive staining for p16*^INK^*^4*a*^ in non-proliferative cells (Ki-67-negative) (Figure 4A(a–e)), and only caspase-3 activity (double-stained cells) in p16*^INK^*^4*a*^-positive cells (Figure 4A(f–j)). Metabolic activity increased in treated cells from degenerate NP tissue, whereas no difference was found in cells from non-mildly-degenerate tissue. Metabolic activity was 24.54% higher following curcumin and 19.15% higher following o-Vanillin treatment, indicating increased proliferation after the reduction of senescent cells and SASP factor expression (*p* < 0.01) (Figure 4B). Also, caspase 3/7 activity indicated that the number of apoptotic cells increased following treatment in cells from degenerate but not from non-mildly-degenerate tissue. The caspase 3/7 activity was significantly increased by 18.73% (*p* < 0.05) following curcumin treatment and by 18.48% (*p* < 0.01) following o-Vanillin treatment, compared to the untreated control, in cells of degenerate NP tissues, while no change was observed in treated and untreated cells from non-mildly-degenerate tissue (Figure 4C). Moreover, the results from IHC were consistent with the caspase 3/7 and metabolic activity results. Apoptosis was evaluated by fluorescence microscopy, and cells from non-mildly-degenerate tissue showed a similarly low level of apoptosis in treated and untreated cultures (Figure 4D(a–i)). In contrast, cells isolated from degenerate IVDs displayed an increased level of apoptosis when treated with curcumin and o-Vanillin (Figure 4D(m–u)). Hydrogen peroxide was used as a positive control for both non-mildly-degenerate (Figure 4D(j–l)) and degenerate cells (Figure 4D(v–x)). Altogether, the data confirms our hypothesis that curcumin and o-Vanillin are senolytic for senescent IVD cells, thus consolidating their potential as senolytics for IVD degeneration.

#### 3.4.1. Curcumin and o-Vanillin Reduced Senescent Cells in Pellet Culture

**Figure 5:**
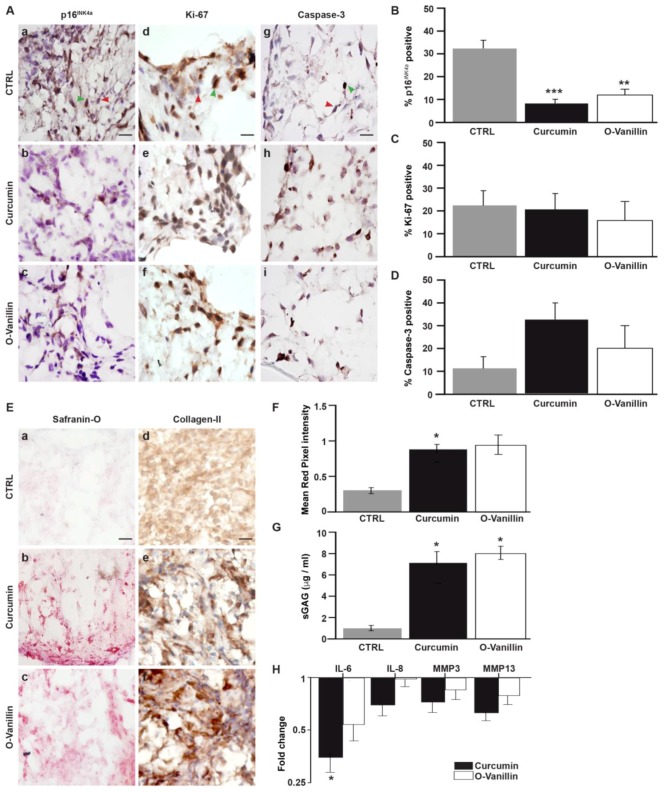
Evaluation of extracellular matrix production in pellet cultures. (**A**) Representative images of p16*^INK4a^* (a–c), Ki-67 (d–f), and caspase-3 (g–i) staining. Head arrows indicate positive (green) and negative (red) staining. Quantification of (**B**) p16*^INK4a^* expression, (**C**) Ki-67, and (**D**) caspase-3. (**E**) Representative images of degenerate NP pellets stained for safranin-O (a–c) and collagen type II (d–f). (**F**) Overall safranin-O intensity and (**G**) sulfated glycosaminoglycan (sGAG) release quantification. (**H**) Conditioned media was analyzed with ELISA for SASP factors, and the results are presented as fold-changes compared to the control. *n* = 5, Scale bars: 100 μm in (**A**) and 200 μm in (**E**). Values are presented as mean ± SEM in (**B**–**D)** and (**F**–**H**). * indicates a significant difference, *p* < 0.05; ** *p* < 0.01 and *** *p* < 0.001.

A pellet culture of IVD cells has been suggested to more closely mimic 3-dimensional in vivo conditions, and it allows for the evaluation of the matrix synthesis. We first verified a non-toxic range of curcumin and o-Vanillin in pellet cultures from degenerate tissue, using the Alamar Blue assay. As in the monolayer, no cytotoxicity was observed below 50 μM for curcumin, or at any of the o-Vanillin concentrations, while curcumin concentrations at 75–200 μM reduced cell activity (*p* = 0.21) (Figure S3C). Four days of treatment reduced the protein expression of p16*^INK^^4a^* (Figure 5A(a–c)); 77% (*p* < 0.001) of p16*^INK^^4a^*-positive cells were cleared by curcumin and 62% (*p* < 0.01) by o-Vanillin (Figure 5B). Proliferation rates were comparable, as revealed by Ki-67 staining (*p* = 0.7 and *p* = 0.88) (Figure 5A(d–f) and (Figure 5C). The number of apoptotic cells was higher in the treated cultures (Figure 5A(g–i)), with an 18% (*p* = 0.077) and 10% (*p* = 0.084) increase in caspase-3 positive cells in curcumin and o-Vanillin-treated pellets, respectively (Figure 5D). Collectively, these results further support a potential senolytic effect by curcumin and o-Vanillin in cells of degenerating human IVDs.

#### 3.4.2. Curcumin and o-Vanillin Positively Affect Matrix Synthesis

An increase in proteoglycan content (Figure 5E(a–c)) and a trend for increased collagen type II staining was observed following treatment (Figure 5E(d–f)). Proteoglycan intensity increased, with 59% (*p* < 0.05) and 36% (*p* = 0.12) in cell pellets from degenerate tissue exposed to curcumin and o-Vanillin, respectively (Figure 5F). Treatment also significantly increased sGAG release into the culture media, with 7.27 μg/mL in curcumin-, and 8 μg/mL in o-Vanillin-treated cultures, compared to 0.957 μg/mL in vehicle-treated controls (*p* < 0.05) (Figure 5G). The increase in proteoglycan and collagen type-II content following treatment further suggests that the removal of senescent cells promotes matrix synthesis. Protein levels of the SASP-associated factor IL-6 were significantly decreased in curcumin- (3-fold) (*p* < 0.05) and o-Vanillin (2-fold) (*p* = 0.055) treated groups, compared with the control. IL-8 (*p* = 0.09 and *p* = 0.86), MMP3 (*p* = 0.2 and *p* = 0.64) and MMP13 (*p* = 0.16 and *p* = 0.14) secretion also decreased after exposure to both compounds (Figure 5H). Together, the data indicate that curcumin and o-Vanillin promote matrix synthesis and decrease SASP factor release.

### 3.5. NFkB and Nrf2 Expression Is Higher in Degenerate Discs Compared to Non-Mildly-Degenerate Discs

**Figure 6:**
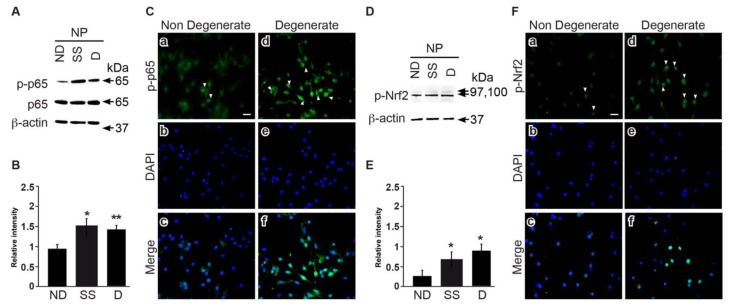
NFkB and Nrf2 expression in degenerate NP cells. NFkB and Nrf2 phosphorylation and nuclear translocation was evaluated by (**A**,**D**) immunoblotting and (**C**,**F**) immunocytochemistry in degenerate and non-mildly-degenerate cells. D: degenerate, ND: non-mildly-degenerate, SS: surgical samples. Densitometry data of the phosphorylated protein is normalized to total p65 (**B**) and β-actin, for Nrf2 (**E**). β-actin was used as a loading control. (*n* = 3 to 5). Scale bars: 20 μm in (**C**,**F**). Values are presented as mean ± SEM in (**B**,**E**). * indicates a significant difference of *p* < 0.05, and ** of *p* < 0.01.

The high phosphorylation levels and nuclear translocation of NFkB and Nrf2 are indicative of an inflammatory environment. Here, we compared cells from non-mildly-degenerate (ND) and degenerate (D) IVDs of organ donors with cells from degenerate surgical samples (SS). Degenerate cells from surgical samples and organ donors revealed higher p- NFkB levels (*p* < 0.05 and *p* < 0.01) (Figure 6A,B), and immunocytochemistry indicated increased NFkB nuclear translocation (Figure 6C) in cells from degenerate IVDs than in cells from non-mildly-degenerate tissue. A similar pattern was observed for Nrf2, which showed higher phosphorylation levels (*p* < 0.05) (Figure 6D,E) and nuclear translocation (Figure 6F) in cells from degenerate IVDs.

### 3.6. Curcumin and o-Vanillin Modulate Inflammatory Signaling Pathways

**Figure 7:**
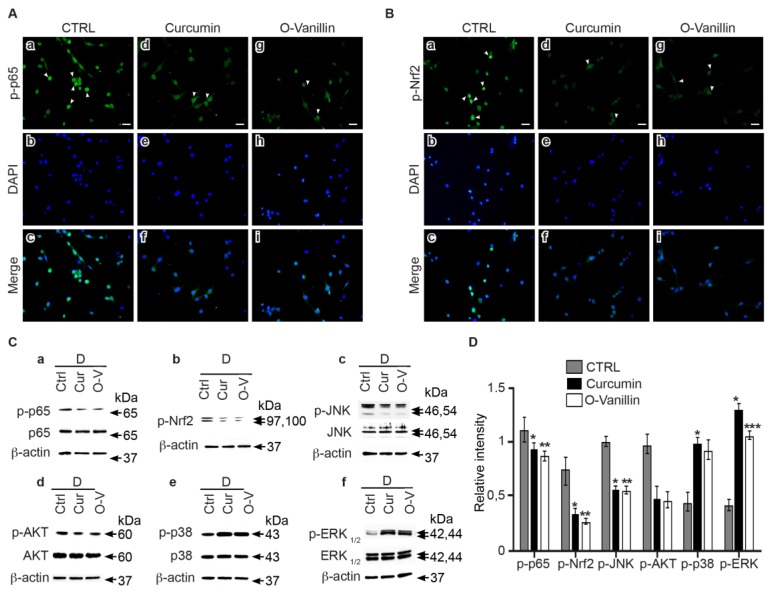
Curcumin and o-Vanillin modulate inflammatory signaling pathways. The effects of curcumin and o-Vanillin treatment on NFkB (**A**) and Nrf2 (**B**) nuclear translocation were confirmed by immunocytochemistry in NP cells of degenerate IVDs. (**C**) Evaluation of the effects of curcumin and o-Vanillin treatment on p65, Nrf2, JNK, AKT, p38, and ERK1/2 phosphorylation, by immunoblotting (a–f) and densitometry quantification (**D**). Activity was evaluated by using phosphorylated-specific antibodies and antibodies recognizing total proteins. β-actin was used as a loading control. (*n* = 3 to 5). Scale bars: 20 μm in (**A**,**B**). Values are presented as mean ± SEM in (**D**). * indicates a significant difference of *p* < 0.05; ** of *p* < 0.01, and *** of *p* < 0.001.

Curcumin- or o-Vanillin-treated or untreated NP cells from degenerate IVDs were stained with anti-NFkB and anti-Nrf2 antibodies, and fluorescence was detected. We found that treatment decreased NFkB (Figure 7A) and Nrf2 (Figure 7B) nuclear translocation in NP cells from degenerate IVDs. Western blot analysis confirmed a downregulation of NFkB (Figure 7C(a)) and Nrf2 (Figure 7C(b)) phosphorylation following curcumin (*p* < 0.05) and o-Vanillin (*p* < 0.01) treatment (Figure 7D). Decreases in JNK (*p* < 0.05 and *p* < 0.01) (Figure 7C(c),D) and AKT phosphorylation levels (*p* = 0.058 and *p* = 0.07) (Figure 7C(d),D) were also observed, following exposure to curcumin and o-Vanillin. In contrast, p38 (*p* < 0.05 and *p* = 0.07) (Figure 7C(e),D) and ERK1/2 (*p* < 0.05 and *p* < 0.001) (Figure 7C(f),D) phosphorylation were notably up-regulated following treatment. Collectively, these results suggest that curcumin and o-Vanillin mediate their senolytic effects via the Nrf2 pathway, and they reduce SASP factor secretion through the down-regulation of the NFkB pathway.

## 4. Discussion

Senescent cells were long thought to be passive bystanders, but recent data suggest that they actively participate in age-related diseases such as osteoarthritis, heart disease and cancer. Here, we show that curcumin and its metabolite o-Vanillin have senolytic and senomorphic effects on senescent cells of degenerating human IVDs. Reducing the number of senescent cells promoted the proliferation of the remaining cells and generated a more favorable and less inflammatory environment with a reduced SASP. Glucose concentration in the culture media can affect cellular senescence, and standard cell culture media containing 4.5 g/L glucose is often used to culture IVD cells, but it has previously been shown to induce senescence in rat IVD and notochordal cells [32]. Here, we found a higher number of senescent cells following culture in 4.5 g/L than in 2.25 g/L glucose. Thus, 2.25 g/L glucose was used in all subsequent experiments to minimize culture-induced senescence.

While cell cycle arrest is a hallmark of senescence, not all non-dividing cells are senescent, and different markers could give different results. We determined how p16*^INK4a^* and SA-β-gal correlate in the detection of positive cells and found significant differences in the exact number of cells labeled as senescent with the two markers. However, both methods indicated a similar fold change (~40%), with higher levels of senescent cells from degenerate compared to non-mildly-degenerate IVDs. SA-β-gal staining indicated a higher level of positive cells but it has been suggested to also identify quiescent cells in addition to truly senescent cells [33]. This marker may therefore overestimate the number of senescent cells. p16*^INK4a^* staining, on the other hand, could identify a sub-population of senescent cells, thereby underestimating the total number. However, independent of the exact number of cells detected as senescent, our results corroborate with other studies relating cellular senescence to IVD degeneration [34]. In addition, the higher number of senescent cells from degenerating tissue was associated with an increase in SASP factor production.

Extrinsic factors like age, sex and lifestyle are known to affect cell senescence, confounding a direct link to IVD degeneration. Thus, we quantified the number of senescent cells and SASP factor expression from degenerate and non-mildly-degenerate discs of the same individual. Radiographic [29] and macroscopic [30] grading systems were used to define the overall degree of degeneration prior to cell isolation. Interestingly, both degenerate NP and AF tissues had significantly more senescent cells and a higher expression of key genes encoding the senescence marker p16*^INK4a^*, ECM-degrading enzymes (MMP3 and MMP13), and pro-inflammatory cytokines (IL6 and IL8). These results directly correlate with IVD degeneration, with the accumulation of senescent cells and SASP factors in human IVD. To our knowledge, this is the first study to evaluate the basal level of senescent cells in IVDs, with different degrees of degeneration from the same individual.

We found a higher percentage of p16*^INK4a^*-positive cells in non-mildly-degenerate tissue from donors aged 62 ± 14.68 years, compared to younger (40.42 ± 17.23 years) donors. However, the accumulation was clearly higher in degenerate tissue, independent of age. Our findings were in accordance with previous reports suggesting that ageing promotes cell senescence [34].

Curcumin and o-Vanillin are well-known for their anti-oxidative and anti-inflammatory properties. We used 5 μM curcumin and 100 μM for o-Vanillin; the concentrations were selected based on their effects on metabolic activities and on concentrations used in previous publications. In our experiments, curcumin concentrations above 50 µM were toxic. Interestingly, similar and higher concentrations of curcumin were not cytotoxic in mixed population of IVD cells [35]. Furthermore, o-Vanillin (200 μM) showed no cytotoxicity in our cultures, corroborating with previous studies [36]. Treatment with both compounds reduced the number of senescent cells and SASP factors, and it was accompanied with an increase in cell proliferation. Curcumin has been shown to activate caspase-3, and to induce time- and dose-dependent apoptosis in cancer cells [37]. To evaluate if curcumin and o-Vanillin are senolytic to senescent IVD cells, we next investigated whether the two compounds increased proliferation (Figure 3D) and apoptosis (Figure 3E), by selectively killing off the non-proliferating senescent cells, or by increasing the proliferation and apoptosis of non-senescent cells. Cells from degenerate (with about 30% of the cells were p16*^INK4a^*-positive cells) and non-mildly-degenerate (6% were p16*^INK4a^*-positive cells) IVDs were evaluated. Our results demonstrate that both compounds selectively induce apoptosis in senescent (p16*^INK4a^* positive) but not proliferating (Ki-67 positive) cells (Figure 4A). Moreover, curcumin and o-Vanillin increased metabolic activity (Figure 4B), caspase 3/7 activity (Figure 4C), and apoptosis (Figure 4D) in cells from degenerate IVDs, but not in cells from non-mildly-degenerate IVDs. These results suggest senolytic potential, where curcumin and o-Vanillin are killing off senescent cells isolated from human IVDs.

IVD cells maintain their native phenotype better when cultured in a three-dimensional culture system, and it allows for matrix synthesis to be evaluated [38]. Collecting all isolated cells in pellets also reduces the risk of selection bias, based on their adhesion to plastic culture dishes. In pellet cultures, we found a more profound reduction in the senescent cells following treatment, which could be explained by a higher number of cells being captured using this method. The different times of treatment for the monolayer and pellet cultures could also play a role. The short course of treatment (6 h) in monolayer cultures resulted in a rapid reduction in senescent cells, reinforcing the hypothesis of senolytic activity. In contrast, the longer treatment (4 days) and the following culture (21 days) gave both compounds more time to suppress senescence markers, and/or prevent secondary senescence, adding to the senolytic and senomorphic effects consistent with their anti-inflammatory properties [11].

The loss of proteoglycan and decreased collagen type II production are regarded as typical pathological changes in IVD degeneration. Interestingly, curcumin and o-Vanillin significantly increased the proteoglycan contents of both NP cell pellets and the surrounding culture media. Both compounds also increased collagen type-II production and reduced the secretion of SASP factors. These results suggest a comparable effect of the two compounds and highlight the beneficial effects of curcumin and o-Vanillin on IVD health, and they are potential therapeutics for IVD degeneration.

Curcumin has been reported to act on multiple cellular targets and signaling pathways in cells of different origins [39]. NFkB and Nrf2 signaling pathways are both important in the defense mechanism against oxidative stress and cellular senescence [40]. Our findings demonstrate higher expression and nuclear translocation of p65 and Nrf2 in cells from degenerate IVDs. Both decreased following treatment with curcumin and o-Vanillin. The lower level of NFkB nuclear translocation indicates a downregulation of inflammatory gene transcription. Also, the decrease in Nrf2 expression highlights the homeostatic condition where Nrf2 is maintained at low levels by the inhibitory factor Keap1 [41]. These results corroborate with reported data that curcumin modulates the Nrf2-Keap1 complex, leading to Nrf2 binding to the antioxidant responsive element [42] and the translocation inhibition of NFkB into the nucleus [43].

The dysregulation of multiple signaling pathways has been shown to be implicated in IVD degeneration. The transcription factor Nrf2 is activated by upstream kinases, including PI3K/AKT, JNK/SAPK, ERK1/2, p38, and NFkB [44]. Therefore, major MAPK pathways were also investigated following treatments with curcumin and o-Vanillin. JNK activation triggers senescence [45], and a link between JNK/SAPK and p16*^INK4a^* has been reported [46]. Interestingly, our results demonstrate a decrease of JNK activation in the treated cells, suggesting that this pathway is a mediator of the senolytic effect.

PI3K/AKT plays a critical role in IVD degeneration [47], and silencing of AKT induces the apoptosis of human-degenerated NP cells [48]. We found a decrease in the phosphorylation levels of AKT in the treated groups, suggesting that curcumin and o-Vanillin mediate apoptosis through AKT inhibition. Curcumin modulation of the AKT pathway has been reported in cancer cells, and a crosstalk between the AKT and NFkB signaling pathways [49] has been described. Both the ERK and p38 pathways were activated in NP cells when they were exposed to curcumin and o-Vanillin. ERK1/2 phosphorylation has been described to stimulate IVD cell proliferation [50]. Although p38-MAPK is reported to mediate senescence in fibroblasts [51], an increase in p38 in human NP has also been linked to IVD cell proliferation. Indeed, p38, which is upstream of Nrf2, can both stimulate and inhibit Nrf2 nuclear translocation.

To date, few natural compounds have been shown to display senolytic activity. The data presented here suggest that curcumin and o-Vanillin may be used as senolytic and anti-inflammatory drugs for senescent IVD cells. The presented observations prompt the need for further investigations of the two compounds and their therapeutic contributions in reducing cellular senescence and retarding IVD degeneration in vivo.

## Figures and Tables

**Table 1:** Characteristics of the donors utilized for the study. (-): AF and NP cells were used as indicated. (ICC): Immunocytochemistry including SA-β-gal, p16*^INK4a^*, Ki-67, caspase-3 staining, and immunofluorescence for p65 and Nrf2 in a monolayer culture. (IHC): Immunohistochemistry for p16*^INK4a^*, collagen type II, and Safranin-O in pellet culture sections. (RT-qPCR): Real-time Quantitative Polymerase Chain Reaction (WB): Western blot (ELISA): Enzyme-linked immunosorbent assays (DMMB): Dimethyl methylene blue (DMMB) assays.

Donor	Age	Sex	Cause of Death	ICC	IHC	RT-qPCR	WB	ELISA	DMMB
1	84	F	Motor vehicle accident	-		-			
2	15	M	Motor vehicle accident	-	-				
3	49	M	Unknown	-	NP	-		NP	NP
4	42	M	Unknown	-	-				
5	28	M	Motor vehicle accident	-	-				
6	50	M	Anoxia	-	-	-			
7	68	M	Cerebral hemorrhage	-			NP		
8	41	M	Cerebral anoxia				NP		
9	44	M	Anoxia	-	NP	NP		NP	NP
10	53	F	Anoxia Carbon Monoxide	-	NP	-		NP	NP
11	27	M	Trauma	-					
12	51	M	Unknown	-	NP	NP		NP	NP
13	66	F	Head trauma						
14	60	F	Hemorrhage/ischemic	-	-	-			
15	34	M	Unknown		-				
16	55	F	Anoxia		-				
17	49	F	Arachnoid hemorrhage			-			
18	53	M	Subarachnoid hemorrhage	-					
19	53	M	cerebral aneurysm rupture	-	NP	-	NP	NP	NP
20	62	M	Anoxia	-					
21	76	F	Anoxia	NP	NP	-	NP	NP	NP
22	52	M	Cerebral vascular accident	NP		-	NP		
23	17	M	Brain death	NP	-	NP	NP		
24	67	M	Hemorrhagic stroke			NP			
25	73	F	Cerebrovascular accident			NP			
26	68	F	Cerebral hemorrhage			NP			
27	39	M	Gunshot to the neck						
28	35	F	Surgical sample	-		-	NP		
29	30	F	Surgical sample	-		-	NP		
30	32	F	Surgical sample	-		-	NP		
31	45	M	Surgical sample	-					
32	27	M	Surgical sample	-					
33	38	F	Surgical sample	-		-			
34	36	F	Surgical sample	-		-			
35	47	F	Surgical sample	-		-			

**Table 2:** Real time-PCR oligonucleotide primers list.

Target	Forward Primer Sequence	Reverse Primer Sequence	Ref
**p16*^INK4a^***	5′- CTGCCCAACGCACCGAATA-3′	5′-GCTGCCCATCATCATGACCT-3′	[26]
**IL6**	5′-TGAACCTTCCAAAGATGGCTG-3′	5′-CAAACTCCAAAAGACCAGTGATG-3′	[26]
**IL8**	5′-TCCTGATTTCTGCAGCTCTG-3′	5′-GTCTTTATGCACTGACATCTAAGTTC-3′	[26]
**MMP3**	5′-AATGGCATTCAGTCCCTCTATG-3′	5′-GACAGGTTCCGTGGGTAC-3′	[26]
**MMP13**	5′-GATGACGATGTACAAGGGATCC-3′	5′-AGGGTCACATTTGTCTGGC-3′	[26]
**GAPDH**	5′-TCCCTGAGCTGAACGGGAAG-3′	5′-GGAGGAGTGGGTGTCGCTGT-3′	[24]

## Supplementary Materials

The following are available online at https://www.mdpi.com/2077-0383/8/4/433/s1, Figure S1: High glucose in culture media increased NP and AF cell senescence, Figure S2: The senescence inducer peroxide was used as positive control, Figure S3: Degeneration levels in IVDs of the same individual.
