# Supplementary material for: Curcumin and o-Vanillin Exhibit Evidence of Senolytic Activity in Human IVD Cells In Vitro
Source: J Clin Med. 2019 Mar 29;8(4):433. doi: 10.3390/jcm8040433 (PMC6518239; doi:10.3390/jcm8040433)
Supplement: Supplementary file 1 [file jcm-08-00433-s001.pdf]

Supplementary Materials:

Figure S1

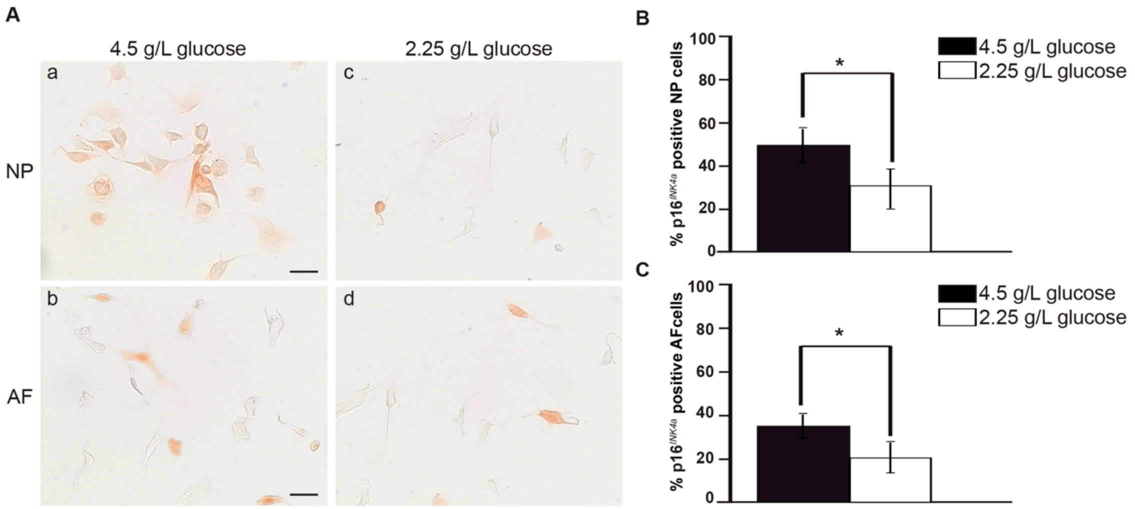

Fig. S1. High glucose in culture media increased NP and AF cell senescence (A) Representative photomicrographs of p16^{INK4a} positive NP and AF cells cultured in media containing 4.5 **(a-b)** or 2.25 **(c-d)** g/L glucose. **(B-C)** Quantification of p16^{INK4a} positive NP **(B)** and AF **(C)** cells (n= 5, Average age= 61.8 ±19.47). Scale bars: 25μm **(A)**. Values are presented as the mean ±SEM percentage of p16^{INK4a} positive cells. * indicates a significant change; $p < 0.05$.

Figure S2

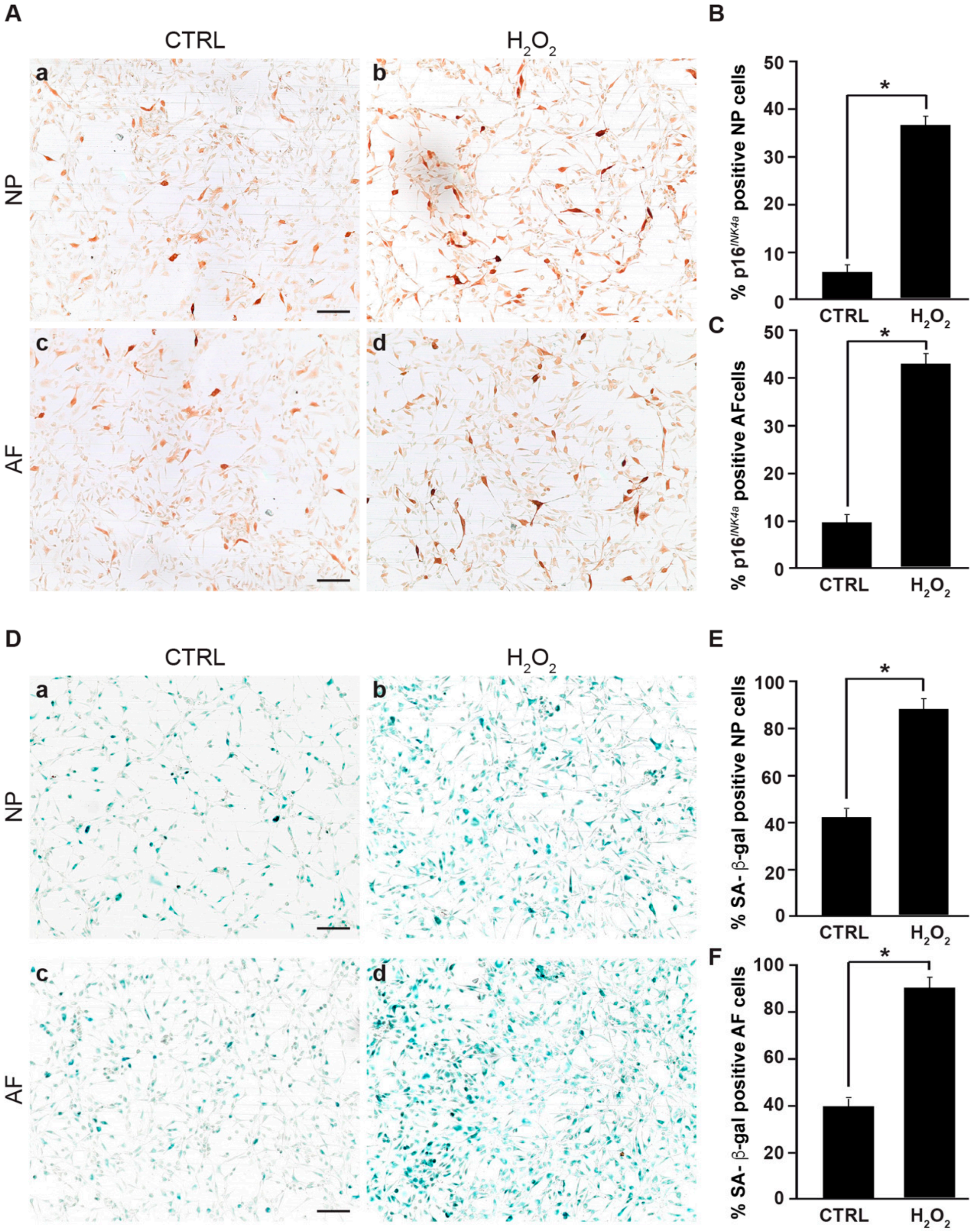

Fig. S2. The senescence inducer peroxide was used as positive control. (A) Representative photomicrographs of p16*INK4a* expression with or without peroxide (H_2O_2) (50µM) for 6h. Quantification of p16*INK4a* positive NP **(B)** and AF **(C)** cells. **(D)** Representative photomicrographs of SA-β-gal activity and quantification in **(E)** NP and **(F)** AF cells. Scale bars: 10µm **(A)**. Values are presented as mean ±SEM. * indicates a significant change; $p < 0.05$.

Figure S3

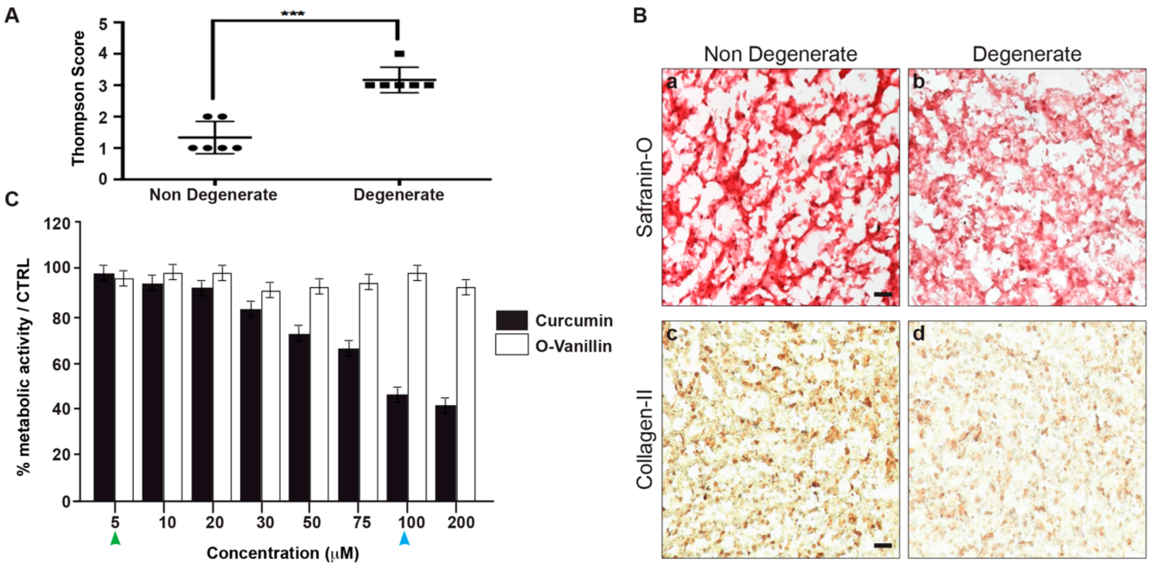

Fig. S3. Degeneration levels in IVDs of the same individual. (A) Representation of the degeneration grade for 12 IVDs from 6 individuals. **(B)** Representative images of Safranin-O staining **(a-b)** and collagen type II **(c-d)** expression in degenerate and non-mildly-degenerate NP tissue from the same individual. **(C)** Viability of degenerated d-NP cells in pellet culture exposed to Curcumin and O-Vanillin was evaluated with Alamar blue assay and presented in fold change compare to the control (n=4). Scale bars: 200µm **(B)**. n=6 and average age= 62 ±14.68. Values are presented as mean ±SEM. *** indicates a significant difference; $p < 0.001$.
